# In ovo feeding of nicotinamide riboside affects broiler pectoralis major muscle development^1,2^

**DOI:** 10.1093/tas/txaa126

**Published:** 2020-07-16

**Authors:** John M Gonzalez, Ashunti R Jackson

**Affiliations:** 1 Department of Animal and Dairy Science, University of Georgia, Athens, GA; 2 Cobb-Vantress, Inc., Siloam Springs, AR

**Keywords:** avian, development, muscle fiber, nicotinamide riboside, pectoralis major

## Abstract

The objective of this study was to examine the effect of nicotinamide riboside (NR) on pectoralis major muscle (PM) development and growth. Fertilized Cobb 500 broiler eggs (*N* = 156; average weight of 70.3 g) were ordered by weight, and within each four egg strata, eggs were randomly assigned to treatments within a 2 × 2 factorial arrangement. Factor 1 consisted of NR treatment with eggs receiving 0 or 250 mM NR. Factor 2 consisted of injection location, with treatments injected into either the yolk sac or albumen. Eggs were incubated at a temperature of 37 °C and a relative humidity of 40 ± 2% for the first 18 d of incubation and humidity was increased to 60 ± 2 °C for the final 3 d. On day 10 of incubation, eggs were injected in their designated location with 100 µL of 0.9% sterile saline containing the assigned NR dose. Chicks were hatched, euthanized, and morphometric measurements of the body and left PM were collected. The left PM was also analyzed for muscle fiber cross-sectional area (CSA) and density. There were no treatment × location or main effects for all body morphometric measurements (*P* > 0.07), except chest width of chicks from eggs injected in the yolk were wider (*P* = 0.01) than chicks from eggs injected in the albumen. There were only treatment × location interactions for PM weight and length (*P* < 0.01). When NR was injected into the albumen, PM weight did not differ (*P* = 0.09); however, when NR was injected into the yolk sac, PM weight increased (*P* < 0.01). When NR was injected into both locations, PM length increased (*P* < 0.01), but increased to a greater extent when NR was injected into the yolk sac. There were treatment main effects for PM width and depth (*P* < 0.01), with NR injected chicks having PM with greater width and depth. There were no treatment × location or main effects for PM fiber CSA (*P* > 0.06). There was a treatment × location interaction (*P* < 0.01) for fiber density. When NR was injected into the albumen, fiber density did not differ (*P* = 0.09); however, when NR was injected into the yolk sac, fiber density increased (*P* < 0.01). Injecting NR into the yolk sac of the developing embryo at day 10 of incubation increased PM development which was due to an increase in muscle density.

## INTRODUCTION

Similar to red meat species, poultry muscle fiber number is determined during prenatal development. Although red meat species’ offspring in utero muscle development is heavily influenced by maternal factors (Greene et al., 2019; Gauvin et al., 2020), poultry have no such influence once the egg is laid. Although developing chicken embryos are not influenced by their hens, numerous studies stated nutrients within the egg were not sufficient to support maximal growth (Ohta et al., 1999; Zielinska et al., 2011). This led scientists to explore in ovo feeding as a means to supply extra nutrients.

In ovo feeding is the practice of injecting compounds into various locations within the incubating egg. Initially, the technology was developed for vaccine administration (Sharma and Burmester, 1982). In the literature, groups examined the effects of injecting compounds including growth hormone (Hargis et al., 1989; Kocamis et al., 1999), amino acids (Ohta et al., 2001; Bhanja et al., 2008), and insulin-like growth factor-1 (IGF-1; Liu et al., 2012; Wang et al., 2012) on muscle development. Others groups also demonstrated nutrient delivery using nanoparticles such as diamond (Grodzik et al., 2013), platinum (Prasek et al., 2013), silver (Sawosz et al., 2013), and gold (Zielinska et al., 2011) affected muscle development. The literature does not indicate why the industry has not widely adopted these technologies, the fact that some compounds are toxic or subject to extra regulations may provide an explanation for lack of adoption. Therefore, identifying compounds that improve muscle development but are considered natural can be beneficial to the industry.

Nicotinamide riboside (NR), a new pyridine-nucleoside form of vitamin B3, occurs naturally at low concentrations in yeast, bacteria, and milk (Chi and Sauve, 2013). In mouse embryonic stem cells and young and aged mouse models, NR supplementation increased nicotinamide adenine dinucleotide (NAD^**+**^) levels (Yang et al., 2007, Zhang et al., 2016). Rathbone et al. (2009) reported sirtuin-1, a major consumer of NAD^+^, increased muscle satellite cell proliferation, and Zhang et al. (2016) also found NR increased both NAD^+^ and satellite cell number in aged mice. Because NR influenced satellite cell number through increased NAD^+^ production, the compound could positively influence the events of chicken embryonic muscle growth and development. The hypothesis of the experiment was in ovo feeding of NR to the developing broiler embryo will increase pectoralis major (PM) muscle mass. The objective of this study was to examine the effects of NR in ovo feeding on broiler PM growth and development.

## MATERIALS AND METHODS

The methodology outlined below was approved by the Kansas State University Institutional Animal Care and Use Committee (Approval # 3816.1).

### Egg Procurement, Incubation, and Injections

Fertilized broiler eggs (*N* = 156; Cobb 500; Cobb-Vantress, Siloam Springs, AR) with an average weight of 70.3 g were transported in coolers to the Kansas State University Muscle Biology Laboratory (Manhattan, KS). Upon arrival, egg weights were recorded, eggs were ordered by weight, and within each four egg strata, eggs were randomly assigned to treatments within a 2 × 2 factorial arrangement. Factor 1 was NR treatment with eggs receiving 0 or 250 mM NR (ChromaDex, Los Angeles, CA). Factor 2 consisted of injection location, with treatments injected into either the yolk or albumen (Albumen 0 *n* = 27; Albumen 250 *n* = 32; Yolk 0 *n* = 30; Yolk 250 *n* = 24). After treatment assignment, eggs were positioned with equal treatment representation onto trays and placed in an incubator (Sportsman 1502; GQF Manufacturing Company Inc., Savannah, GA) set to operate at a temperature of 37 °C and a relative humidity of 40 ± 2% for the first 18 d of incubation. The incubator rotated hourly to reposition eggs and trays were rotated daily throughout the incubator to account for variation in temperature and humidity. Tray weights were recorded each day to determine egg weight loss percentage with a target weight loss of 0.67% per day.

### Injection Procedure

At day 10 of incubation, NR with the equivalent weight of 250 mM was added to 0.9% sterile saline and covered with foil to prevent exposure to light. Sets of 20 eggs representing equal treatment numbers were removed from the incubator and candled to determine location of the yolk and albumen, and the injection site was cleaned with 70% ethanol. Eggs were turned at a 90° angle and a 2.54-cm, 20-guage hypodermic needle was used to create an opening in the shell at the proper injection site. The needle was inserted approximately 1 cm into injection site and 100 µL of the 250 mM NR solution or 0.9% saline solution was injected into the egg. A 1-cm^2^ portion of medical tape (Nexcare; 3M, Maplewood, MN) was positioned over the injection location and eggs were returned to the incubator.

### Hatching, Euthanasia, and Processing

On day 18 of incubation, the relative humidity of the incubator was increased to 60 ± 2% and eggs were placed into hatching boxes at the bottom of the incubator. As chicks began to hatch, they were removed from the incubator, marked for treatment, and relocated to a box positioned underneath a heat lamp. Within 24 h after hatch, chicks were euthanized by prolonged exposure to CO_2_ gas and decapitation. Chick weights were recorded, and digital calipers (Traceable Digital Calipers; Fisher Scientific, Pittsburg, PA) were utilized to measure crown to rump length, head width, and head length. Head and chest circumference were also collected by wrapping a string around the designated area and determining the length against a ruler.

Chick carcasses were sprayed with 70% ethanol and the skin and feathers were pulled back to reveal the PM muscles. Prior to PM muscles removal, chest width and length were measured using digital calipers. The left and right PM muscles were removed, careful to not remove the PM muscles. The left PM was weighed, and dimensions were collected using digital calipers, including length, width, and depth. This muscle was positioned onto a tongue depressor and placed into a −80 °C freezer, where it was stored until it was used for cryosectioning. The heart and liver were also removed, weighed, and discarded.

### Immunohistochemistry

The methods of Noel et al. (2016) were followed for immunohistochemistry with modifications. The PM was removed from the tongue depressor, embedded in tissue embedding media (Fisher Scientific), cooled with isopentane chilled liquid nitrogen, and stored at −80 °C until cryosectioning. Ten micrometer-thick cryosections were cut using a Microm 550 cryostat (Thermo Fisher Scientific, Kalamazoo, MI), and six cryosection were collected on positively charged slides (Diamond White Glass; Globe Scientific Inc., Paramus, NJ). Cryosections were incubated in a blocking solution containing 5% horse serum and 0.2% TritonX-100 in phosphate-buffered saline (PBS) for 30 min. Slides were incubated for 1 h at room temperature in a primary antibody solution consisting of blocking solution and 1:500 rabbit, α-dystrophin (Thermo Fisher Scientific). Cryosections were washed three times for 5 min each in PBS and incubated for an additional 30 min with blocking solution containing 1:1,000 Alexa-Flour 594 goat-anti-rabbit heavy and light chains (Life Technologies, Carlsbad, CA). Cryosections were washed again as stated above, 5 µL of 9:1 glycerol in PBS was placed on each section, and slides were coverslipped for imaging. Cryosections were visualized at 200× magnification using a Nikon Elipse TI-U inverted microscope (Nikon Instruments Inc., Melville, NY), a Nikon DS-QiMC digital camera (Nikon Instruments Inc.) was used to photograph cryosections, and an average of 1,000 fibers were analyzed using NIS-Elements imaging software (Basic Research, 3.3; Nikon Instruments Inc.) to determine fiber cross-section area (CSA).

### Statistics

Data were analyzed as a completely randomized design with a 2 × 2 factorial arrangement and egg as the experimental unit. Nicotinamide riboside treatment (TRT) and injection location (LOC) and their interaction served as fixed effects. The PROC MIXED procedure of SAS 9.4 (SAS Inst. Inc., Cary, NC) was utilized, and pairwise comparisons between the least square means were computed using the PDIFF option of the LSMEANS statement. Differences were considered statistically significant at *P* < 0.05.

## RESULTS

There were no TRT × LOC interactions for all measures (*P* > 0.07; Table 1), except PM weight and length (*P* < 0.01). Pectoralis major weights of chicks injected with NR in the albumen were not different (*P* = 0.09) when compared with chicks injected with saline in the albumen; however, chicks injected with NR in the yolk had greater (*P* < 0.01) PM weights than those injected with saline. Pectoralis major lengths of chicks injected with NR in the albumen were longer (*P* = 0.04) than those injected with saline. Lengths of chicks injected with NR in the yolk were longer (*P* < 0.01) than those injected with saline, but the difference was greater than the albumen response.

**Table 1.** Body and pectoralis major morphometrics of hatched chicks injected in ovo with nicotinamide riboside during embryogenesis

Nicotinamide riboside dose^1^	0 mM		250 mM			*P*-value^2^		
Injection location^3^	Albumen	Yolk	Albumen	Yolk	SEM	TRT	LOC	TRT × LOC
*n*	27	32	30	24				
Weight, g	42.69	42.99	43.19	44.02	6.40	0.17	0.31	0.63
Body measurements								
Dimensions, mm								
Crown-rump length	95.60	96.93	96.14	89.36	3.70	0.12	0.23	0.07
Head width	15.40	15.74	15.56	15.45	0.40	0.67	0.48	0.21
Head length	21.79	21.86	21.29	21.73	0.19	0.42	0.51	0.63
Head circumference	32.38	32.08	32.14	31.49	1.60	0.70	0.66	0.87
Chest length	22.01	21.96	21.98	22.68	0.55	0.36	0.39	0.29
Chest width	16.90	17.35	16.57	17.69	0.33	0.98	0.01	0.28
Heart weight, g	0.36	0.36	0.35	0.36	0.02	0.75	0.55	0.24
Liver weight, g	1.09	1.08	1.05	1.11	0.06	0.64	0.36	0.19
Pectoralis major measurements								
Weight, g	0.13^a^	0.13^a^	0.14^a^	0.18^b^	0.01	<0.01	<0.01	<0.01
Dimensions, mm								
Length	17.55^a^	17.04^a^	18.68^b^	20.74^c^	0.43	<0.01	0.06	<0.01
Width	4.59	4.62	4.81	5.21	0.22	<0.01	0.13	0.20
Depth	2.27	2.32	2.43	2.65	0.09	<0.01	0.09	0.30

^abc^Treatments with different superscripts within a row differ (*P* < 0.05). ^1^100 µL of 0.9% saline containing 0 or 250 mM nicotinamide riboside injected at day 10 of incubation. ^2^TRT denotes nicotinamide riboside treatment main effect; LOC denotes injection location main effect. ^3^Treatments were injected into either the yolk sac or albumen of the egg.

Treatment did not affect whole-body or organ measures (*P* > 0.12); however, NR treatment did increase PM weight, length, width, and depth (*P* < 0.01). There were no LOC main effects for all measures (*P* > 0.06), with the exception of an increased chest width and PM weight when injection took place in the yolk (*P* = 0.01).

**Figure 1.**
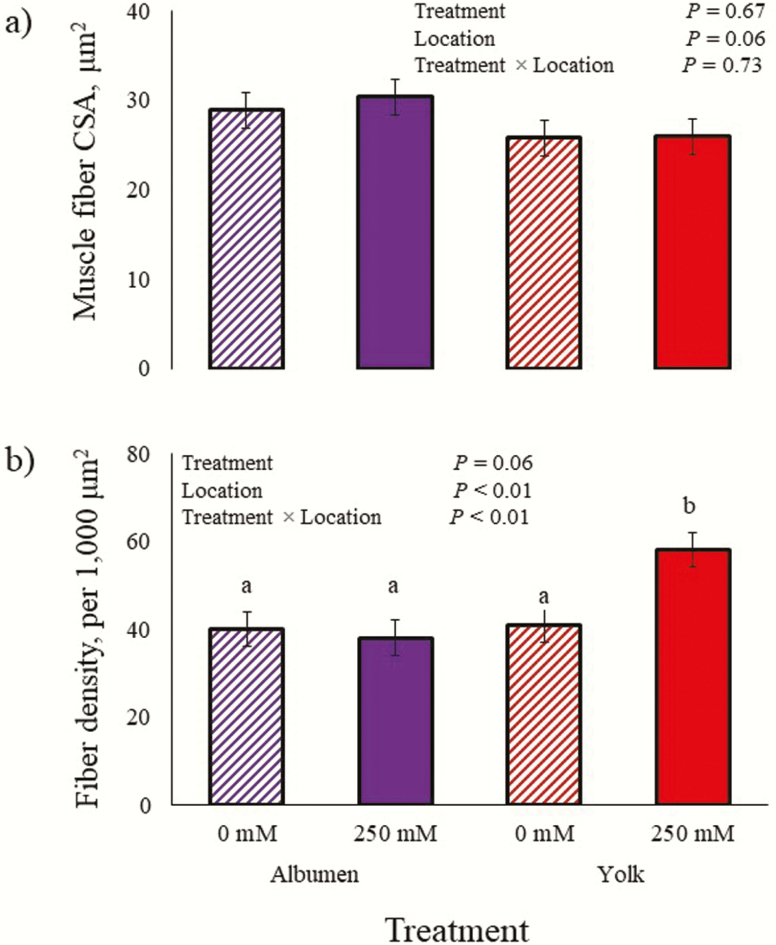
(a) Muscle fiber cross-sectional area (CSA) and (b) density of hatched chicks fed in ovo with nicotinamide riboside (NR) during embryogenesis. At day 10 of incubation, embryos were injected with 100 µL of 0.9% saline containing 0 or 250 mM NR in the yolk sac or albumen. Immunohistochemistry and muscle fiber morphometric data collection were conducted as described by Noel et al. (2016). ^a,b^Means with different superscripts differ (*P* < 0.05).

There were no TRT × LOC interaction or LOC and TRT main effects for muscle fiber CSA (*P* > 0.06; Fig. 1). There was a TRT × LOC interaction for muscle fiber density (*P* < 0.01). Chicks injected with NR in the albumen did not differ (*P* = 0.09) in fiber density when compared with chicks injected with saline in the albumen; however, chicks injected with NR in the yolk had more (*P* < 0.01) muscle fibers than those injected with saline. The TRT main effect did not affect (*P* = 0.06) muscle fiber density, but chicks injected in the yolk had more (*P* < 0.01) fibers than chicks injected in the albumen.

## DISCUSSION

Of the major protein-producing industries, the broiler industry has made the greatest advancement in production efficiency and yield. Siegel (2014) reported over a 25-yr period ending in 2010, broiler market weight increased almost 75% on only 0.44 kg more feed. Genetic selection and advancements in nutrient utilization are factors responsible for improved production efficiency (Buzala and Janicki, 2016). Aside from efficiency of nutrient utilization, the most notable improvement in broiler production is the amount of deposited carcass muscle. Although not commonly practiced in industry, several researchers have explored in ovo feeding as a means to provide extra nutrients to maximize avian growth during embryogenesis. In the current study, in ovo feeding of NR did not affect any whole-body morphometric measurements or heart and liver weight. Opposite to the findings of the current study, Al-Murrani (1982) and Ohta et al. (2001) found in ovo feeding of amino acids in the yolk sac increased embryo and hatched chick weights. Zhao et al. (2017) also reported hatched chick body weight increased when creatine pyruvate was fed to developing embryos in the amnion. l-Carnitine is a supplement that produces NAD^+^ similar to the salvage pathway NR operates through. Similar with the current study, in ovo injection of l-carnitine, into the amnion of the developing embryo at day 18 did not affect hatched chick body weight (Zhai et al., 2008). These findings indicate improvement in whole-body morphometrics may be dependent on the compound injected into the egg.

Even though whole-body measures were unaffected by NR injection, administration of NR into the yolk sac increased PM weight and length by 38% and 22%, respectively. Regardless of injection location, NR also increased PM width and depth by 9% and 11%, respectively. No other studies have examined the effect of NR on broiler embryo development; however, Liu et al. (2012) reported IGF-1 injected into the albumen of 12-d incubated duck embryos increased 27-d embryo and 2-d post-hatchling PM weight by 5.5% and 5.8%, respectively. Kocamis et al. (1998) found broilers injected with IGF-1 at days 15 or 16 of incubation had larger day 42 post-hatch breast muscles 19% and 17%, respectively. Uni et al. (2005) reported day 17 incubated embryos fed a cocktail to maintain glucose homeostasis increased hatched chick PM weight by 10% and this advantage was extended to 15% by day 25 of post-hatch growth. Despite not measuring PM weight at hatch, Zhao et al. (2017) found chickens in ovo fed creatine pyruvate had greater PM weights at days 21 and 42 post-hatch by 21% and 13%, respectively. When compared with these studies, the NR treatment administered in the present study had a greater effect on PM development and growth and these advantages may be increased as the bird grows post-hatch.

The two major events of broiler embryo muscle development include primary muscle fiber formation, followed by secondary muscle fiber formation (Lawrence et al., 2012). Primary myogenesis in the avian embryo encompasses days 3 to 7 of incubation and secondary myogenesis occurs from day 8 until hatch (Chal and Pourquie, 2017). In the current study, in ovo feeding occurred at day 10 of incubation which is well into the events of secondary myogenesis. Feeding NR at this time period did not affect PM fiber CSA, but injecting NR into the yolk sac increased PM fiber density by 45%. While Zhao et al. (2017) did not measure muscle fiber number or density, the authors found feeding creatine pyruvate increased post-hatch day-21 and -42 PM fiber CSA by 30% and 14%, respectively. Liu et al. (2012) demonstrated injecting IGF-1 into developing duck embryos did not affect breast muscle CSA or fiber density, but injections increased leg muscle CSA and density when embryos were harvested 1 d prior to hatch. Zielinska et al. (2012) reported injecting gold nanoparticles and taurine together or separately increased PM cell number by a maximum of 43%, and only injecting a combination of the molecules increased fiber diameter by 37%. Therefore, the NR response documented in the current study elicited the greatest increase in PM fiber density of known in ovo feeding studies, which caused the increased PM weight.

## IMPLICATIONS

Nicotinamide riboside is a novel vitamin B_3_ analog that has not been extensively utilized in poultry production. Because NR increases NAD^+^ production in tissues and the sirtuin-1 protein regulates stem cell activity in response to NAD^+^ levels, this is the possible mechanism by which NR increased PM weight and fiber density. Currently, it is unknown if the increased PM weight stimulated by in ovo NR feeding will affect future growth and meat quality; however, the NR response could affect the poultry industry yields, product quality, or incidence of myopathys; all which are future areas of research.

## References

[CIT0001] Al-Murrani W. K. 1982 Effect of injecting amino acids into the egg on embryonic and subsequent growth in the domestic fowl. Br. Poult. Sci. 23:171–174. doi:10.1080/00071688208447943 7074386

[CIT0002] Bhanja S. K., Mandal A. B., Agarwal S. K., Majumdar S., and Bhattacharyya A.. 2008 Effect of in ovo glucose injection on the post-hatch growth, digestive organ development and blood chemical profiles in broiler chickens. Indian J. Anim. Sci. 78:869–872.

[CIT0003] Buzala M., and Janicki B.. 2016 Review: effects of different growth rates in broiler breeder and layer hens on some productive traits. Poult. Sci. 95:2151–2159. doi:10.3382/ps/pew173 27194733

[CIT0004] Chal J., and Pourquie O.. 2017 Making muscle: Skeletal myogenesis in vivo and in vitro. Development 144:2104–2122. doi:10.1242/dev.151035 28634270

[CIT0005] Chi Y., and Sauve A. A.. 2013 Nicotinamide riboside, a trace nutrient in foods, is a vitamin B3 with effects on energy metabolism and neuroprotection. Curr. Opin. Clin. Nutr. Metab. Care 16:657–661. doi:10.1097/MCO.0b013e32836510c0 24071780

[CIT0006] Gauvin M. C., Pillai S. M., Reed S. A., Stevens J. R., Hoffman M. L., Jones A. K., Zinn S. A., and Govoni K. E.. 2020 Poor maternal nutrition during gestation in sheep alters prenatal muscle growth and development in offspring. J. Anim. Sci. 98:1–15. doi:10.1093/jas/skz388 PMC6981092 31875422

[CIT0007] Greene M. A., Britt J. L., Powell R. R., Feltus F. A., Bridges W. C., Bruce T., Klotz J. L., Miller M. F., and Duckett S. K.. 2019 Ergot alkaloid exposure during gestation alters: 3. Fetal growth, muscle fiber development, and miRNA transcriptome1. J. Anim. Sci. 97:3153–3168. doi:10.1093/jas/skz153 31051033 PMC6606534

[CIT0008] Grodzik M., Sawosz F., Sawosz E., Hotowy A., Wierzbicki M., Kutwin M., Jaworski S., and Chwalibog A.. 2013 Nano-nutrition of chicken embryos. The effect of in ovo administration of diamond nanoparticles andl-glutamine on molecular responses in chicken embryo pectoral muscles. Int. J. Mol. Sci. 14:23033–23044. doi:10.3390/ijms141123033 PMC3856104 24264045

[CIT0009] Hargis P. S., Pardue S. L., Lee A. M., and Sandel G. W.. 1989 In ovo growth hormone alters growth and adipose tissue development of chickens. Growth Dev. Aging 53:93–99. 2599745

[CIT0010] Kocamis H., Kirkpatrick-Keller D. C., Klandorf H., and Killefer J.. 1998 In ovo administration of recombinant human insulin-like growth factor-I alters postnatal growth and development of the broiler chicken. Poult. Sci. 77:1913–1919. doi:10.1093/ps/77.12.1913 9872596

[CIT0011] Kocamis H., Yeni Y. N., Kirkpatrick-Keller D. C., and Killefer J.. 1999 Postnatal growth of broilers in response to in ovo administration of chicken growth hormone. Poult. Sci. 78:1219–1226. doi:10.1093/ps/78.8.1219 10472850

[CIT0029] Lawrence T., Fowler V., and Novakofski J.. 2012 Muscle tissue. In: T. Lawrence et al., editor, Growth of farm animals. Oxfordshire (UK): CABI; p. 74–90.

[CIT0012] Liu H. H., Wang J. W., Zhang R. P., Chen X., Yu H. Y., Jin H. B., Li L., Han C. C., Xu F., Kang B., et al. 2012 In ovo feeding of IGF-1 to ducks influences neonatal skeletal muscle hypertrophy and muscle mass growth upon satellite cell activation. J. Cell. Physiol. 227:1465–1475. doi:10.1002/jcp.22862 21618537

[CIT0028] Noel J. A., Broxterman R. M., McCoy G. M., Craig J. C., Phelps K. J., Burnett D. D., Vaughn M. A., Barstow T. J., O’Quinn T. G., Woodworth J. C., et al. 2016 Use of electromyography to detect muscle exhaustion in finishing barrows fed ractopamine-HCl. J. Anim. Sci. 94:2344–2356. doi:10.2527/jas2016-0398 27285911

[CIT0013] Ohta Y., Kidd M. T., and Ishibashi T.. 2001 Embryo growth and amino acid concentration profiles of broiler breeder eggs, embryos, and chicks after in ovo administration of amino acids. Poult. Sci. 80:1430–1436. doi:10.1093/ps/80.10.1430 11599701

[CIT0014] Ohta Y., Tsushima N., Koide K., Kidd M. T., and Ishibashi T.. 1999 Effect of amino acid injection in broiler breeder eggs on embryonic growth and hatchability of chicks. Poult. Sci. 78:1493–1498. doi:10.1093/ps/78.11.1493 10560819

[CIT0015] Prasek M., Sawosz E., Jaworski S., Grodzik M., Ostaszewska T., Kamaszewski M., Wierzbicki M., and Chwalibog A.. 2013 Influence of nanoparticles of platinum on chicken embryo development and brain morphology. Nanosc. Res. Lett. 8:251. doi:10.1186/1556-276X-8-251 PMC3664603 23705751

[CIT0016] Rathbone C. R., Booth F. W., and Lees S. J.. 2009 Sirt1 increases skeletal muscle precursor cell proliferation. Eur. J. Cell Biol. 88:35–44. doi:10.1016/j.ejcb.2008.08.003 18922599 PMC2656762

[CIT0017] Sawosz F., Pineda L., Hotowy A., Jaworski S., Prasek M., Sawosz E., and Chwalibog A.. 2013 Nano-nutrition of chicken embryos. The effect of silver nanoparticles and ATP on expression of chosen genes involved in myogenesis. Arch. Anim. Nutr. 67:347–355. doi:10.1080/1745039X.2013.830520 23952606

[CIT0018] Sharma J. M., and Burmester B. R.. 1982 Resistance to Marek’s disease at hatching in chickens vaccinated as embryos with the turkey herpesvirus. Avian Dis. 26:134–149. 6284106

[CIT0019] Siegel P. B. 2014 Evolution of the modern broiler and feed efficiency. Annu. Rev. Anim. Biosci. 2:375–385. doi:10.1146/annurev-animal-022513-114132 25384148

[CIT0020] Uni Z., Ferket P. R., Tako E., and Kedar O.. 2005 In ovo feeding improves energy status of late-term chicken embryos. Poult. Sci. 84:764–770. doi:10.1093/ps/84.5.764 15913189

[CIT0021] Wang G., Liu H., Li L., and Wang J.. 2012 Influence of ovo injecting IGF-1 on weights of embryo, heart and liver of duck during hatching stages. Int. J. Poult. Sci. 11:756–760. doi:10.3923/ijps.2012.756.760

[CIT0022] Yang T., Chan N. Y., and Sauve A. A.. 2007 Syntheses of nicotinamide riboside and derivatives: effective agents for increasing nicotinamide adenine dinucleotide concentrations in mammalian cells. J. Med. Chem. 50:6458–6461. doi:10.1021/jm701001c 18052316

[CIT0023] Zhai W., Neuman S. L., Latour M. A., and Hester P. Y.. 2008 The effect of male and female supplementation ofl-carnitine on reproductive traits of white leghorns. Poult. Sci. 87:1171–1181. doi:10.3382/ps.2007-00325 18493008

[CIT0024] Zhang H., Ryu D., Wu Y., Gariani K., Wang X., Luan P., D’Amico D., Ropelle E. R., Lutolf M. P., Aebersold R., et al. 2016 NAD^+^ repletion improves mitochondrial and stem cell function and enhances life span in mice. Science 352:1436–1443. doi:10.1126/science.aaf2693 27127236

[CIT0025] Zhao M. M., Gong D. Q., Gao T., Zhang L., Li J. L., Lv P. A., Yu L. L., Gao F., and Zhou G. H.. 2017 In ovo feeding of creatine pyruvate increases hatching weight, growth performance, and muscle growth but has no effect on meat quality in broiler chickens. Livest. Sci. 206:59–64. doi:10.1016/j.livsci.2017.10.013

[CIT0026] Zielinska M., Sawosz E., Grodzik M., Balcerak M., Wierzbicki M., Skomial J., Sawosz F., and Chwalibog A.. 2012 Effect of taurine and gold nanoparticles on the morphological and molecular characteristics of muscle development during chicken embryogenesis. Arch. Anim. Nutr. 66:1–13. doi:10.1080/1745039x.2011.644918 22397092

[CIT0027] Zielinska M., Sawosz E., Grodzik M., Wierzbicki M., Gromadka M., Hotowy A., Sawosz F., Lozicki A., and Chwalibog A.. 2011 Effect of heparan sulfate and gold nanoparticles on muscle development during embryogenesis. Int. J. Nanomed. 6:3163–3172. doi:10.2147/IJN.S26070 PMC3254262 22238506

